# Analysis of Personal and Family Factors in the Persistence of Attention Deficit Hyperactivity Disorder: Results of a Prospective Follow-Up Study in Childhood

**DOI:** 10.1371/journal.pone.0128325

**Published:** 2015-05-29

**Authors:** Ana Miranda, Carla Colomer, M. Inmaculada Fernández, M. Jesús Presentación, Belén Roselló

**Affiliations:** 1 Departamento de Psicología Evolutiva y de la Educación, Universidad de Valencia, Valencia, Spain; 2 Departamento de Educación, Universidad Jaume I, Castellón, Spain; 3 Departamento de Psicología Evolutiva, Educativa, Social i Metodología, Universidad Jaume I, Spain; National Center of Neurology and Psychiatry, JAPAN

## Abstract

**Objectives:**

To study the course of ADHD during childhood and analyze possible personal and family predictor variables of the results.

**Method:**

Sixty-one children with ADHD who were between 6 and 12 years old at the baseline assessment were evaluated 30 months later (mean age at baseline: 8.70 ± 1.97; mean age at follow-up: 10.98 ± 2.19). Status of ADHD in follow-up was identified as persistent (met DSM-IV-TR criteria according to parents’ and teachers’ ratings), contextually persistent (met ADHD criteria according to one informant, and there was functional impairment) and remitted ADHD (with subthreshold clinical symptomatology). Associated psychological disorders of the three groups were analyzed in the follow-up with the *Conners' Rating Scales*. The groups were compared on ADHD characteristics (symptoms of ADHD and impairment), child psychopathology, executive functioning (EF; inhibition, working memory) and parenting characteristics (parental stress and discipline styles) at baseline.

**Results:**

At the follow-up, 55.7% of the children continued to meet the DSM-IV-TR criteria for ADHD, 29.5% showed contextual persistence, and 14.8% presented remission of the disorder. The persistent and contextually persistent ADHD groups showed more associated psychological disorders. Inattention, oppositional problems, cognitive problems and impairment at baseline distinguished the remitted ADHD children from the persistent and contextually persistent ADHD children. Moreover, the persistent groups had significantly more emotional liability and higher parental stress than the group in remission, while no differences in EF where found among the groups.

**Conclusions:**

ADHD children continue to present symptoms, as well as comorbid psychological problems, during adolescence and early adulthood. These findings confirm that persistence of ADHD is associated with child psychopathology, parental stress and impairment in childhood.

## Introduction

Currently, follow-up studies show the persistence of the attention deficit hyperactivity disorder (ADHD) in the transition period from childhood to early adolescence, with a variation in the percentages ranging from 45%-55% [1–3], to 70% [4–8] and even 80% [9–11]. The observed variability in the diagnostic stability may be due to the criteria used to determine the diagnostic status (remission at the syndrome, symptom or functional level), the informants used as referents (parents, teachers or both), the time elapsed between baseline and follow-up, the specific age range and sex of the participants, or the type and intensity of the treatments implemented.

In addition to its chronic nature, another worrisome issue is the high comorbidity that characterizes the course of ADHD. More than half of the children with an ADHD diagnosis continue to present other disorders, particularly behavior and anxiety disorders [12–14] and impairments in their academic [15], social [4], and family [16] functioning. In adolescence, even more serious problems usually appear, such as high rates of delinquency [17], risky sexual behaviors [18], or substance abuse [19]. ADHD persistence also negatively affects adaptive functioning in adult life in a variety of domains such as work, academics, domestic responsibilities, partner relationships and community activities [20–22].

This alarming situation has stimulated research on the determinants of the course of ADHD. Genetic studies show the relationship between the DRD4 [23,24], DAT1 [25] and 5-HTTLPR genes and the persistence of the disorder [26]. Moreover, patients with persistent ADHD exhibit more network disturbances than the remitted ADHD group [27], while the best clinical results are related to morphologically intact cortical structures that support the development of more efficient cognitive control [28].

In addition to the biomarkers, the explanatory socio-ecological ADHD models highlight the contribution of environmental factors to the behavioral expression and different developmental trajectories of the disorder [29]. The basic symptomatology at the time of the diagnosis, the level of hyperactivity/impulsivity [3,6], psychiatric comorbidity [9,30], and, especially, behavior problems [2,31] have been shown to influence the persistence and more unfavorable course of ADHD over time.

In recent years, another research area has involved analyzing the relationship between executive functioning (EF) and the course of ADHD, with mixed research results. A systematic review based on eighteen studies concluded that persisters and remitters did not differ on higher or lower level neurocognitive functions, although both had worse performance than controls [32]. These findings do not support the Halperin and Schulz model [33], which suggests a maturation of more consciously controlled neurocognitive functions in ADHD remitters. However, other studies have offered support for the model by showing differences on neuropsychological measures between remittent and persistent subjects at the follow-up [34,35]. When EF were studied longitudinally (not only at the follow-up), no significant relationship was found between improvements in EF and ADHD symptomatology over time [36,37].

Regarding factors related to the family context, a recent review that focused on the relationship between parenting and functional impairments showed that ADHD is associated with greater stress within the family, higher rates of parental psychopathology, and conflicted parent—child relationships [38]. Recent findings confirmed that, compared to controls, parents of children screening positive for ADHD reported greater stress and more inconsistent and hostile parenting [39,40]. Furthermore, the parenting style also seems to have an influence on ADHD persistence. Adolescents with and without persistent ADHD experience more impaired family processes than adolescents without ADHD. However, the adolescents with persistent ADHD report more authoritarian control from their mothers and have more behavioral problems at home than adolescents with non-persistent ADHD [1].

The research by the Biederman and Faraone group is of particular relevance in examining ADHD persistence, as they include different types of factors in their analyses. The researchers followed boys and girls with ADHD from childhood to adulthood. In adolescence (4-year follow-up) they found significant differences between the remittent and persistent groups at baseline, not only on child factors (inattention, high rates of comorbidity with behavior disorders, anxiety and mood, as well as lower levels of psychosocial and academic functioning), but also on the psychosocial and family adversity of the disorder [9]. In the 11 years follow-up [30], the dysfunctionality related to ADHD, maternal psychopathology, and psychiatric comorbidity at baseline, but not the basic symptoms, predicted the persistence of ADHD in adulthood.

In summary, many children with ADHD remain symptomatic. Understanding what characterizes this persistent ADHD group and identifying factors associated with differential developmental outcomes have important repercussions in the clinical setting. The early identification of children who are likely to have a worse course of the disorder will make it possible to perform interventions using more appropriate strategies.

Hence, the present study, which is part of a longitudinal study that followed the course of children with an ADHD diagnosis, proposed two specific objectives. The first was to study the ADHD course during childhood, determining the rates of children who presented persistent, contextually persistent and remitted ADHD, and analyze possible differences among the groups on associated psychological disorders. The second objective was to compare the persistent, contextually persistent and remitted groups on baseline measures related to ADHD characteristics (ADHD symptoms and impairment), child psychopathology, executive functions (verbal working memory, visuo-spatial working memory and inhibition) and parenting characteristics (parental stress and dysfunctional discipline). We hypothesized that the different outcomes would be influenced by the effects of personal and family risk characteristics. Based on the proposals of a multifactorial model, we hypothesized that the persistent groups, especially if the persistence was cross-contextual, would have more severe ADHD symptoms and related child psychopathologies, greater impairments in executive functions, and worse results on parenting measures at baseline than the ADHD remitted group.

## Methods

### Participants

In the present follow-up study, the participants were 61 children with a clinical diagnosis of combined-subtype ADHD and their families, who formed part of the sample of Spanish families from the International Multicenter ADHD Genetic Project (IMAGE) [41], a genetics study of 1400 families funded by the National Institute of Mental Health.

The participants were evaluated at the beginning of the study, between 2003 and 2005 (baseline), and 30 months later, between 2006 and 2008 (follow-up). To identify the initial sample, the records from the Neuropediatric Service of La Fe Children’s Hospital were reviewed with the help of the service’s clinical psychologist, in order to identify children who had received an ADHD combined subtype diagnosis. In addition, neuro-pediatricians and pediatricians from the regions of Castellon and Valencia were contacted, as well as private specialists and psychology clinics. Through these procedures, a total of 321 possible participants were identified. Of these children, 109 were eliminated due to presenting manifestations of a possible ADHD subtype other than the combined subtype or neurological disorders (mainly epilepsy). When the 212 families were contacted, 147 agreed to participate in the study. In the individualized evaluation performed, children were excluded from the study if they presented an estimated intelligence quotient (IQ) of less than 70, suffered neurological or sensorial damage or motor impairments, or had a diagnosis of schizophrenia or generalized developmental disorder. Finally, 81 children met the strict diagnostic criteria for ADHD combined subtype (ADHD-C) from the revised fourth edition of the Diagnostic and Statistical Manual of Mental Disorders (DSM-IV-TR) [42], based on agreement between a neuro-pediatrician and a clinical psychologist and the information from parent and teacher questionnaires.

Moreover, these 81 children met the requirements for the IMAGE study protocol to confirm the ADHD combined subtype diagnosis using the ADHD-section of the Parental Account of Childhood Symptom (PACS), a semi-structured interview that covers ADHD related behavior in different situations (watching TV, reading, playing alone…). The parents have to rate the frequency or severity of their child’s hyperactivity, inattention and impulsive behaviors [43]. A specific algorithm combined and weighed the rated behavior across situations, finally leading to a dichotomous statement about the presence or absence of the corresponding DSM-IV symptom. Clinical impairment was inferred when at least 12 symptoms exceeded the diagnostic threshold, and this was also verified in the PACS interview (for a thorough description of the diagnostic procedure, see Muller et al [44].

At baseline, the participants were between 6 and 12 years old (mean = 8.70 ± 1.97) and had an equivalent IQ within the ranges of normality (mean IQ = 105.98 ± 16.48), estimated by the sum of the scale scores for Vocabulary and Block Design on the *Wechsler Intelligence Scale for children*- *revised* (WISC-R) [45], using the prorating procedure described by Sattler [46].

Thirty months later the participants were again contacted. Due to the sample loss that often occurs in longitudinal studies, the follow-up included the participation of 61 of the 81 families who had participated at baseline, that is, 75% of the original sample. The other 25% did not participate for different reasons: 9 children (11%) did not participate because of loss of their localization (changes in the family address or contact telephone), and 11 participants declined to attend to the evaluations (14%). There were no differences in baseline ADHD severity between the 61 children who continued in the follow-up and the 20 who did not participate, based on the parents’ (inattention: *t* (79) = -0.90, *p* = .366; hyperactivity-impulsivity: *t* (79) = -0.87, *p* = .389; DSM-total: *t* (79) = -0.60, *p* = .549) and teachers’ ratings (inattention: *t* (79) = 1.12, *p* = .266; hyperactivity-impulsivity: *t* (79) = -0.72, *p* = .476; DSM-total: *t* (79) = 0.37, *p* = .712). There were no differences in other child characteristics assessed at the study baseline either. At follow-up, participants were between 8 and 14 years old (mean = 10.98 ± 2.19). Of the total sample, 58 were boys (95.1%), and 3 were girls (4.9%). A clinical psychologist with more than 10 years of experience, under the supervision of a senior investigator, again evaluated the ADHD symptomatology. Attention was paid to the concordance between parents’ and teachers’ ratings on the DSM-IV-TR criteria for the ADHD diagnosis and information about indicators of the children’s functional impairment, which was collected in the interviews with the family.

Table 1 shows the socio-demographic characteristics of the participants and their families.

**Table 1:** Socio-demographic characteristics of the children and their families.

Characteristics of the children	Baseline (n = 61)	Follow-up (n = 61)	Family characteristics	Follow-up
**Age range**	6–12	8–14	**Psycho-emotional problems mother (% yes)**	29.5
**Mean age**	8.70	10.98	**Family structure**	83.6
(Stan. dev.)	(1.97)	(2.19)	Two parents (%)	
**Sex** (% boys)	95.1	95.1	One-parent (%)	16.4
**Medication** (% Yes)	44.3	70.5*	**Child-rearing figure**	
**Mean IQ**	105.98	-	Mother (%)	62.3
(Stan. dev.)	(16.48)	-	Father (%)	3.3
Associated problems (%)**			Both (%)	32.8
Oppositionism (39–90)	65.6	45.9	Others (%)	1.6
Cognitive problems (47–90)	95.1	82	**Educational level (father/mother)**	
Anxious-shy (40–90)	18	42.6	Lower Secondary (%)	52.5/45.9
Social problems (45–90)	49.2	45.9	Upper Second./Vocational Ed. (%)	31.1/34.4
Emotional lability (41–90)	44.3	47.5	3-year university degree (%)	4.9/11.5
Conners’Global Index (45–90)	95.1	75.4	5-year university degree (%)	11.5/8.2

* % of participants who had taken medication at some point in their lives.

**Associated problems: % who presented a score of T ≥ 63 on the CPRS-RL. The range of the variables are given in brackets (Tscore).

### Measures

#### ADHD characteristics and child psychopathology

*Conners' Rating Scales—Revised*, *long version (CRS—R*:*L*) [47]. This is an instrument validated for children and adolescents between 3 and 17 years old. It collects information about ADHD symptomatology and associated disorders, and has shown acceptable validity and reliability [48]. There are two versions, one for parents (CPRS-R:L) and the other for teachers (CTRS-R:L), containing 80 and 59 items, respectively, rated on a 4-point Likert-type scale (0 for never and 3 for very often). Of the 13 scales contained in the parents’ version, the scales selected for the present study were the oppositional, cognitive problems, anxious/shy, and social problems scales, as well as the emotional lability index, the global index, and the scales related to the DSM-IV-TR for ADHD diagnosis (inattention and hyperactivity-impulsivity). From the teachers’ version, only the three last scales were chosen that included ADHD criteria according to the DSM-IV. The direct scores obtained on each scale were transformed into T scores, which made it possible to objectively and reliably compare the participants according to their age and sex. To identify problematicity, the cut-off point adopted was a T score equal to or greater than 63, equivalent to PC 85.

*Strengths and Difficulties Questionnaire for Parents* (SDQ) [49]. This version, designed for parents of children between 4 and 16 years old, includes two parts: a) 25 items that measure a series of psychological attributes, and b) a parent-rated Impact Supplement that rates functional impairment. This study used the second part, the “impact supplement”, on which the parents rated whether their child had difficulties with emotions, concentration, behavior, or being able to get along with other people, and the degree to which these difficulties were upsetting the child or interfering with the child’s life (home life, friendships, classroom learning, leisure activities or peer relationships). The responses to each of the items are coded between 0 to 3, with 0 representing no impairment and 3 representing extreme impairment in daily life.

#### Executive functions

Three aspects of executive functioning were assessed: verbal working memory, visuo-spatial working memory and inhibition. Verbal working memory was evaluated with the *WISC-R Digit-Span Subtest*, *backward* [45], where the child must repeat a sequence of digits the experimenter has read aloud, but in reverse order. Visio-spatial working memory was evaluated by the *Temporo- Spatial Retrieval Task* (TSRT) [50]. This is a computerized task that consists of 30 trials organized in two phases: a stimuli presentation stage in which the child is asked to pay attention to 12 blue squares distributed randomly on the screen that sequentially change to the color red. In the second response phase, the squares are again presented on the screen, and the child must point to them with his/her finger, following the sequence in which they have changed color. Specifically, the score obtained on the delayed recall condition (where a black screen appears for 1500 msec. between the two phases, increasing the demands on working memory) was used. Finally the *Continuous Performance Test* (CPT) [51] was selected to evaluate inhibition. This study used the computerized CPT-AX version, which has a duration of 8 minutes [52]. On this task, white capital letters are randomly and successively presented every 600 msec. (A, B, F, G, H, J, K, N, T, V, X) at the center of the screen on a black background. The child must respond by pressing the space bar on the computer keyboard as fast as he/she can every time he/she sees an X preceded by an A, which occurs 50 times. The indicator used consisted of the errors of commission (pressing the space bar when only the A appears, or only the X, or any other stimulus different from the AX association).

#### Parenting characteristics

*The Parenting Stress Index* (PSI) [53] is a 126-item parent-report measure of parents’ perceived stress. The PSI includes both the parent domain (which includes depression, attachment, restricted role, competence, isolation, spouse support and health) and the child domain (which includes distractibility/hyperactivity, adaptability, reinforces parent, demandingness, mood, and acceptability).

Parents rate each item on a 5-point Likert scale (1 = strongly disagree to 5 strongly agree). The scores are added up to obtain a total stress score that assesses the overall magnitude of parental stress. Scores from the PSI have acceptable internal consistency (α = 0.95) and test-retest reliability (r = 0.71–0.82) [53]. In the present study, parents’ scores on the total stress scale were used.

*The Parenting Scale* [54]. This 30-item questionnaire measures parents’ dysfunctional discipline styles by asking them about the probability of the parent using specific discipline strategies. For each item, on a 7-point Likert scale, the parents have to choose the number that best describes the parenting style with their child during the past 2 months. The scale yields a total score and three factors: Laxness (permissive, inconsistent discipline); Over-reactivity (displays of anger, meanness and irritability); and Verbosity (lengthy verbal responses or reliance on talking). High scores on the total discipline scale reflect a general style of dysfunctional discipline practices.

The scale has adequate reliability (0.84) and discriminates between parents of clinical and non-clinical children. It correlates with self-report measures of child behavior, marital discord and depressive symptoms, and also with observational measures of dysfunctional discipline and child behavior [54].

### Ethics and procedure

At baseline, participants with a diagnosis of combined subtype ADHD were selected, and behavioral (ADHD symptoms, associated psychological disorders and impairment), neuropsychological (inhibition and verbal and visuo-spatial working memory) and family (discipline style and parental stress) measures were collected.

The follow-up evaluation took place a mean of 30 months after the initial evaluation. After giving the participants a complete description of the study, written informed consent was obtained from the parents and the children, based on the procedures approved by the clinical research ethical committee (*Hospital La Fe* and *Ministerio de Educación y Ciencia* of Spain). To make sure treatment did not mask the experimental results, all the children had their medication withdrawn 48 hours before the sessions.

### Statistical analysis

In the follow-up phase, the participants were categorized in three groups taking into account the information provided by the parents and teachers based on the DSM-IV-TR criteria for ADHD (any of the subtypes): 1) subjects with a persistent status, when both informants positively rated the presence of 6 or more criteria from the DSM-IV- TR; 2) subjects with a contextually persistent status, who met the ADHD criteria according to the ratings of one evaluator, whether parents or teachers, and had social and/or academic impairments, recorded as affirmative responses to the SDQ-Impact Supplemental questions “Do you think your child has difficulties in one or more of the following areas: emotions, concentration, behavior or being able to get on with other people?”, “Do the difficulties upset or distress your child?”, and interference in at least one of the child's everyday life areas (home life/friendships/classroom learning/leisure activities); and 3) subjects with a remission status, who did not meet criteria for the disorder according to either the parents’ or teachers’ ratings.

Data analysis was conducted with the SPSS statistical package, version 19.00. To compare the groups’ associated psychological disorders in the follow-up, Multivariate Analysis of Covariance (MANCOVA) was used. Four MANCOVAs were conducted to compare the data obtained at baseline (ADHD characteristics, child psychopathology, executive functions and parenting characteristics) among the subjects with remission, persistence, or a status of contextual persistence of ADHD. The age of the participants was included as a covariate to avoid interpreting the differences due to the broad age range of the participants as effects of development. Post-hoc analysis with Bonferroni correction was performed. The degrees of freedom are not the same in all the MANCOVAs, due to the lack of data from some participants on the neurocognitive tests and on the family variables. The proportion of total variance accounted for by the independent variables was calculated using the partial eta squared (according to Cohen [55]: eta squared, .06 = small; .06-.14 = medium, .14 = large).

## Results

### Persistence of ADHD and associated psychological disorders at follow-up

**Fig 1:**
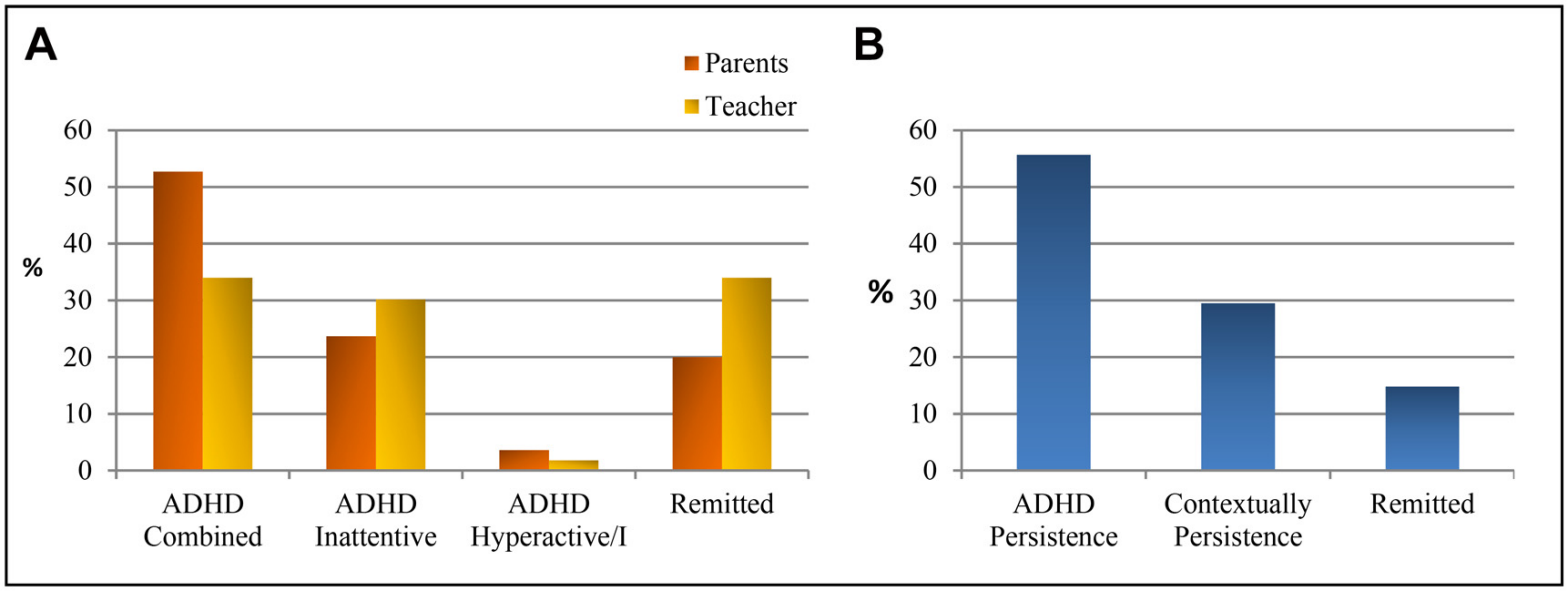
ADHD persistence at follow-up. **1a**) Percentage of participants who meet the DSM-IV-TR criteria for ADHD or are in remission at the follow-up, according to parents’ and teachers’ ratings. **1b**) Percentage of participants who meet the DSM-IV-TR criteria for ADHD, taking into account the criterion of cross-situational persistence: persistent (met the DSM-IV-TR criteria with agreement on parents’ and teachers’ ratings), contextually persistent (met ADHD criteria according to one informant, and there was functional impairment) and remitted ADHD (subthreshold clinical symptomatology).

According to the parents’ ratings (Fig 1a), 52.7% of the children continued to meet the ADHD combined subtype criteria at the follow-up 30 months later, 23.7% had moved to the inattentive subtype, and 3.6% to the hyperactive-impulsive subtype, while 20% no longer met the strict criteria of the DSM-IV-TR for ADHD. According to the teachers’ ratings (Fig 1a), 34% of the children continued to meet the criteria for combined subtype ADHD, 30.2% for the inattentive subtype, 1.8% for the hyperactive-impulsive subtype, and 34% no longer met the diagnostic criteria for ADHD.

Based on the cross-situational nature of the ADHD symptoms and impairment, we defined three mutually exclusive categories of outcomes at the follow-up: persistent, contextually persistent and remitted. According to the parents’ and teachers’ ratings, at the follow-up assessment, 55.7% of the subjects continued to present a diagnosis of ADHD (21.3% combined subtype and 34.4% inattentive subtype). Only 14.8% presented remission of the disorder at the follow-up, meeting less than six inattention and hyperactivity-impulsivity criteria according to the ratings of both parents and teachers. Finally, in the remaining 29.5%, the group with contextual persistence, there was no agreement between the parents’ and teachers’ ratings, so that only one of the evaluators determined that the diagnostic criteria were met for one of the ADHD subtypes, even though the child had a functional impairment (Fig 1b).

The three groups (persistent, contextually persistent and remitted) presented statistically significant differences in associated psychological disorders at the follow-up (Wilks Lambda = .47, F_12,104_ = 3.97, *p* < .001, η^2^_p_ = .314). Specifically, the differences were statistically significant on all the Conners’ variables used, except Emotional Lability: Oppositional (F_2,57_ = 8.31; *p* = .001; η^2^_p_ = .226), Cognitive Problems (F_2,57_ = 18.35; *p* < .001; η^2^_p_ = .392), Anxious/shy (F_2,57_ = 3.70; *p* = .031; η^2^_p_ = .115), Social Problems (F_2,57_ = 6.82; *p* = .002; η^2^_p_ = .193) and Global Index (F_2,57_ = 9.58; *p* < .001; η^2^_p_ = .252). Bonferroni’s post hoc test revealed statistically significant differences between the remitted group and both the persistent and contextually persistent groups on Oppositional (*p* < .001; *p* = .005), Cognitive problems (*p* < .001; *p* = .001), Social Problems (*p* = .001; *p* = .036) and Conners’ Global Index (*p* < .001; *p* = .004). In these cases, the persistent and contextually persistent ADHD groups presented significantly higher scores than the remitted group. On the Anxious/shy variable, only the remitted group showed significantly lower scores than the contextually persistent group (*p* = .042). Finally, the persistent and contextually persistent groups presented significant differences on the Cognitive Problems variable (*p* = .032), as the persistent group showed greater problematicity (Table 2).

**Table 2:** Associated psychological disorders at follow-up: Comparison of Persistent, Contextually Persistent and Remitted ADHD.

	Persistent(n = 34)		Contextually Persistent(n = 18)		Remitted(n = 9)		Statistic		
*Follow-up*	M	SD	M	SD	M	SD	F	η^2^_P_	Group Difference
Oppositional Problems	66.18	10.52	65.33	13.20	51.00	12.85	8.31*	.226	Remitted < Contextually Persistent, Persistent
Cognitive Problems	74.32	6.98	68.89	8.66	57.78	6.14	18.35**	.392	Remitted < Contextually Persistent < Persistent
Anxious/shy	58.06	11.60	66.17	15.50	53.11	11.43	3.70*	.115	Remitted < Contextually Persistent
Social Problems	66.88	13.86	61.67	10.99	48.22	3.80	6.83*	.193	Remitted < Contextually Persistent, Persistent
Emotional Lability	63.82	12.87	65.06	11.64	56.78	19.06	2.02	.066	-
Conners’ Global Index	73.65	9.36	72.39	10.33	60.11	12.81	9.58**	.252	Remitted < Contextually Persistent, Persistent

*p < .05,

**p < .001.

The three groups were also significantly different from each other in the use of pharmacological treatment (generally psycho-stimulants) (χ2 (2) = 7.02, *p* = .030). In fact, 33.3% of the children in the remitted group had taken medication at some point in their lives, while in the case of the persistent and contextually persistent ADHD groups, this percentage rose to 76.5% and 77.8%, respectively. Moreover, 47.1% of the persistent ADHD group consistently took medication during the time period between baseline and follow-up, while the remaining 29.4% of the subjects had taken medication for less time. In the ADHD group with contextual persistence, the rates were 55.6% and 22.2%, respectively. In the remitted ADHD group, 11.1% took medication continuously between baseline and follow-up, and 22.2% for a shorter period of time.

### Comparison of the groups on baseline characteristics

To study the factors that influence the persistence of ADHD, the group with a remitted status, was compared to the group with a contextually persistent and a persistent status on ADHD characteristics, child psychopathology, executive functions and parenting characteristics rated at baseline, using age as covariate.

The results of the first MANCOVA showed that overall there were statistically significant differences among the three groups on baseline ADHD characteristics (Wilks Lambda = .34, F_6,102_ = 4.30, *p* = .001, η^2^_p_ = .202). Specifically, statistically significant differences were found on the variables: inattention (F_2,53_ = 7.60; *p* = .001; η^2^_p_ = .223) and impairment (F_2,53_ = 5.17; *p* = .009; η^2^_p_ = .163), but not on the symptomatology of hyperactivity/impulsivity (Table 3). The remitted group presented statistically significant differences with both the persistent and contextually persistent groups on the Inattention (*p* = .044, *p* = .001) and Impairment variables (*p* = .007, *p* = .037). In both cases, the remitted ADHD had better scores than the other two groups.

**Table 3:** Baseline variables: Comparison of Persistent, Contextually Persistent and Remitted ADHD.

	Persistent		Contextually Persistent		Remitted		Statistic		
*Variables*	M	SD	M	SD	M	SD	F	η^2^_P_	Group Difference
***ADHD characteristics***									
Inattention	72.72	4.89	75.88	5.82	67.33	4.97	7.60**	.223	Remitted < Contextually Persistent, Persistent
Hyperactivity/imp.	79.50	6.95	81.50	5.50	77.78	6.96	1.17	.042	-
Impairment	7.28	2.61	6.81	3.08	3.78	2.68	5.17*	.163	Remitted < Contextually Persistent, Persistent
***Child Psychopathology***									
Oppositional Problems	66.65	11.25	66.28	8.24	54.44	11.47	5.98*	.173	Remitted < Contextually Persistent, Persistent
Cognitive Problems	72.32	5.72	75.78	5.14	65.56	5.00	10.29**	.265	Remitted < Contextually Persistent, Persistent
Anxious/shy	53.06	7.68	55.89	13.19	53.89	9.20	0.49	.017	-
Social Problems	66.12	16.33	68.78	13.31	57.00	13.00	1.62	.054	-
Emotional Lability	63.29	9.86	63.28	7.91	56.00	9.51	3.71*	.115	Remitted < Persistent
Conners’ Global Index	74.62	5.76	75.78	4.14	68.44	7.83	6.20*	.179	Remitted < Contextually Persistent, Persistent
***Executive Functions***									
Digit reverse	3.93	1.21	3.64	1.01	4.14	1.57	0.54	.024	-
Temporo	4.33	1.27	4.57	1.22	4.43	1.13	0.11	.005	-
CPT-commissions	33.26	33.54	28.50	28.18	34.86	31.49	0.28	.009	-
***Parenting***									
Discipline Style	98.56	17.80	100.78	25.03	96.00	22.64	0.51	.032	-
Parenting Stress	313.48	40.99	279.33	40.90	268.56	49.09	6.45*	.239	Remitted < Persistent

*p < .005,

**p < .001.

ADHD characteristics N = 57;Child Psychopathology N = 61; Executive Functions N = 48; Parenting N = 45.

The three groups also showed differences in child psychopathology (Wilks Lambda = .54, F_12,104_ = 3.13, *p* = .001, η^2^_p_ = .265). Specifically, the differences were statistically significant on Oppositional (F_2,57_ = 5.98; *p* = .004; η^2^_p_ = .173), Cognitive Problems (F_2,57_ = 10.29; *p* < .001; η^2^_p_ = .265), Emotional Lability (F_2,57_ = 3.71; *p* = .030; η^2^_p_ = .115) and the Conners Global Index (F_2,57_ = 6.20; *p* = .004; η^2^_p_ = .179), but not on Anxiety and Social Problems (Table 3). Bonferroni’s post hoc test reveals statistically significant differences between the remitted group and both the persistent and contextually persistent groups on Oppositional (*p* = .005; *p* = .001), Cognitive problems (*p* = .007; *p* < .002) and Conners’ Global Index (*p* = .007; *p* = .005). Moreover, the ADHD persistent group presented significantly higher scores on Emotional Lability than the remitted group (*p* = .028).

The MANCOVA conducted to evaluate the differences in the executive functioning variables did not show statistically significant differences (Wilks Lambda = .94, F_6,84_ = 0.46, *p* = .838, η^2^_p_ = .032). The same analyses were performed with the family variables of parenting stress and dysfunctional discipline style, with the results of the MANCOVA showing statistically significant differences (Wilks Lambda = .75, F_4,80_ = 3.15, *p* = .018, η^2^_p_ = .136). The posterior ANCOVAS showed differences on the total stress variable (F_2,41_ = 6.45; *p* = .004; η^2^_p_ = .239), but not on discipline style (Table 3). In this case, the ADHD group with a persistent status showed worse scores than the ADHD remitted group (*p* = .006).

## Discussion

This longitudinal study had two specific aims. The first objective consisted of providing estimates of the course of ADHD and mapping its correlates over time. The second objective was to identify risk factors at baseline associated with differential outcomes of persistence/remission, related to the psychopathology of the child, EF and parenting.

Our data support the “diagnostic stability” of ADHD. Thus, at the follow-up, nearly 60% of the children continued to meet the diagnostic criteria for one of the subtypes of the disorder, while only 14.8% could be assigned the status of remitted. Moreover, when adding the contextually persistent category, the percentage rose to 85.2%, similar to the 78% found by Biederman et al [30] in an 11-year follow-up study. Furthermore, examining the percentages of persistence according to the ratings of parents and teachers separately, the rates we find ascend to 80.3% and 67.8%, respectively, agreeing with studies that show a rate of persistence in early adolescence of around 70% [4,7,8]. Overall, these results show the dependence on the specific method used to define the diagnosis, as occurs with estimating the prevalence of ADHD. As reported in a comprehensive meta-analysis, prevalence estimates are considerably lower when different algorithms are used to combine symptom ratings by parents and teachers, from 12.9% of children using the “or-rule” algorithm to 4.0% for raters’ agreements on the ADHD diagnosis [56].

The core manifestations of ADHD changed over time, as 33.9% of the combined subtype participants moved toward the predominantly inattentive subtype. This movement is consistent with the trajectories of the symptoms from early childhood to adolescence [57] and with the tendency observed toward developmental change from the combined subtype to the inattentive subtype [58]. As children get older, hyperactivity-impulsivity (for example, fidgeting, interrupting) tends to decrease more quickly and at an earlier age than inattention (for example, being forgetful or easily distracted) [6,59]. The experience of hyperactivity as a feeling of internal restlessness as the child grows [60] helps the symptoms in this domain to remain under the diagnostic threshold, while inattention will become more evident as school tasks require more attentional capacity. This would justify the higher rate of the predominantly inattentive subtype identified by the teachers (28.8%) compared to the parents (24.6%). These results also coincide with the recent change from the term “subtype” to “presentation” in the DSM 5 to indicate that the ADHD subtypes are unstable over time, reflecting the transition that frequently occurs from one subtype to another during the developmental cycle.

In addition, about 50% of the participants in the persistent and contextually persistent groups took medication continuously, compared to about 10% of the remitted group. This finding suggests that at the follow-up, the greater severity of the symptomatology of the persistent and contextually persistent groups, who present significantly more oppositional problems, cognitive problems, social problems and more general problematicity, has required a prolonged administration of medication over time. In another recent study, more adolescents with persistent ADHD were treated with methylphenidate before and at that time than adolescents with non-persistent ADHD [1].

The persistent and contextually persistent groups present a similar profile of associated psychological disorders in the follow-up. However, there are differences on the level of cognitive problems, even though both groups present more problems than the remitted group. It is necessary to further analyze what occurs with the learning disabilities at these ages, as the persistent group probably presents more severe problems in reading, writing or mathematics than the other two groups.

When the differences in baseline variables among the participants who presented complete persistence, contextual persistence and remitted symptoms were analyzed, the results showed that the two groups with persistence presented a worse profile of child psychopathology variables at baseline; however, no differences were found among the three groups on any of the executive functions evaluated. Thus, the results show the influence of the psychological disorders [2,6,9,61] and the impairment in childhood [30] on ADHD persistence. These results support the idea that indicators of functioning are crucial in evaluating the disorder and designing treatments. In addition, the two persistent groups presented greater inattention at baseline than the remitted group, supporting studies that found more “dreamy symptoms” [61] in adolescents with persistent ADHD.

The family variables were also found to have an important role in the persistence of the disorder. Our results complement the information on the stress experienced by parents of children with ADHD [62], indicating that higher levels of parental stress in childhood are related to the persistence of the disorder. Specifically, the persistent group presented higher parental stress scores than the remission group. This finding may be related to the fact that the children who continued to present ADHD at the follow-up also presented more basic symptoms of the disorder and more comorbidities during childhood. Therefore, these results may be mediated by other variables such as higher levels of oppositionism; future studies could further examine this topic. Another possible explanation is that the parents’ perception that the parental demands on them exceed their resources to cope with them has a direct influence on the development of the disorder. In this case, it would be vital to have intervention programs directed toward parents of children with ADHD, with specific modules on attributional retraining and stress management.

Furthermore, the parents of the children with remitted ADHD did not seem to apply different discipline styles from those reported by parents of children with persistent or contextually persistent ADHD. As other studies have shown, it is likely that parents of children with ADHD in our sample present more dysfunctional discipline styles than parents of children with typical development [63]. Therefore, regardless of the persistence, a childhood ADHD diagnosis was probably related to dysfunctional discipline styles, supporting the bidirectional model. According to this model, the behavioral challenges presented by children with ADHD contribute to reducing the parents’ ability to set limits on inappropriate behaviors and make the usual discipline styles less effective. In turn, the parents’ ineffective disciplining reinforces the child’s maladaptive behavior. However, the results of this study indicate that dysfunctional discipline styles do not seem to be significantly involved in the course or persistence of the disorder itself, although there is evidence of an association between the initial levels of dysfunctional discipline styles and the development, continuation or increase in oppositional and conduct problems in children with ADHD [64–66]. Future studies will have to further examine this question using direct observation of the parent-child interactions.

Finally, the expectation that children with remitted symptoms would show better EF at baseline was not fulfilled. Even though EF impairments in the preschool stage seem to be a good predictor of ADHD symptomatology in the beginning school stage [67], our results indicate that this relationship does not continue until early adolescence. In consonance, other studies have not detected differences in childhood and adolescence in the cognitive performance (inhibition and verbal and visuo-spatial working memory) of children with persistent, remitted and partially remitted forms of the disorder [37], or a significant relationship between an improvement in executive functions and the ADHD symptomatology [36]. However, it should be kept in mind that EF deficits in childhood play a relevant role in the academic problems of adolescents with ADHD [68,69]. Therefore, to obtain a more complete view, it would be beneficial to analyze the association between ADHD persistence/remission and other specific aspects of EF (e.g., planning and organization), in addition to working memory and inhibition.

### Limitations

The limitations affecting this study must be considered. First, the small sample size and the fact that it is composed of children with a clinical diagnosis of combined subtype ADHD may affect the generalization of the results, which cannot be extrapolated to a non-clinical sample. It is also important to note that many of our participants come basically from middle-low class families with no social marginalization; therefore, our findings may be more generalizable to the middle-class. Other limitations have to do with the broad age range of the participants, from 6 to 12 years old in the first evaluation, as well as the long follow-up period, 30 months. It is necessary to replicate the results with more specific age ranges and longer time periods that extend into late adolescence and early adulthood.

The evaluation of executive functioning is another limitation of this study. Although the selection was based on the result of ad hoc reviews that point to inhibition and working memory as the executive functions most involved in ADHD, in future studies it would be advisable to include neuropsychological tasks such as the Stop Signal or the Go/No-go, in order to evaluate other basic ADHD parameters such as performance variability, or contemplate other executive functions such as planning and cognitive flexibility, in addition to using more ecological executive functioning measures.

### Practical implications

The attention deficit with hyperactivity disorder has a chronic nature. A large percentage of children with ADHD continue to present symptoms, as well as comorbid psychological problems, during adolescence and early adulthood. Therefore, understanding the personal and environmental factors that can worsen the severity of ADHD is a particularly critical issue for detecting and planning early efficacious actions that can attenuate the consequences of the disorder throughout the life cycle. For example, guidance programs for parents must go beyond a model based only on medication or behavior modification. Along with these components, parents should be offered information that: reduces their insecurity and feelings of guilt; provides them with strategies to reduce the level of stress that their children’s behavior can cause; teaches them to identify and value progress, even when limited; helps them to promote a clear distribution of responsibilities and roles; and constructs a democratic educational style that is neither coercive nor over-protective [70].

In addition to its great relevance in the clinical setting, as it can help in designing appropriate treatment strategies to speed up remission, better knowledge about the predictors of ADHD persistence will make it possible to concentrate the scant economic resources on the cases with the greatest risk of persistence.

One of the most interesting findings from the present study is that the ADHD cases that become contextually persistent have just as many associated psychological disorders as the cases with complete persistence. Related studies with adults indicate that individuals with partially-remitted ADHD showed similar substance use to those with current ADHD, whereas those in full remission were comparable to normal controls. In summary, although ADHD symptoms may remit with time, individuals who retain persisting or partial symptoms have considerable needs that must be addressed throughout their development.
